# Insulin Sensitivity and Glucose Homeostasis Can Be Influenced by Metabolic Acid Load

**DOI:** 10.3390/nu10050618

**Published:** 2018-05-15

**Authors:** Lucio Della Guardia, Michael Alex Thomas, Hellas Cena

**Affiliations:** 1 Laboratory of Dietetics and Clinical Nutrition Department of Public Health, Experimental and Forensic Medicine, University of Pavia, 27100 Pavia, Italy; Hellas.cena@unipv.it; 2 Department of Biology, Center for Obesity Reversal, Georgia State University, Atlanta, GA 30302, USA; michael.thomas173@gmail.com

**Keywords:** insulin resistance, diabetes, metabolism, metabolic acidosis, glucose homeostasis, dietary acid load, western diet, acid–base

## Abstract

Recent epidemiological findings suggest that high levels of dietary acid load can affect insulin sensitivity and glucose metabolism. Consumption of high protein diets results in the over-production of metabolic acids which has been associated with the development of chronic metabolic disturbances. Mild metabolic acidosis has been shown to impair peripheral insulin action and several epidemiological findings suggest that metabolic acid load markers are associated with insulin resistance and impaired glycemic control through an interference intracellular insulin signaling pathways and translocation. In addition, higher incidence of diabetes, insulin resistance, or impaired glucose control have been found in subjects with elevated metabolic acid load markers. Hence, lowering dietary acid load may be relevant for improving glucose homeostasis and prevention of type 2 diabetes development on a long-term basis. However, limitations related to patient acid load estimation, nutritional determinants, and metabolic status considerably flaws available findings, and the lack of solid data on the background physiopathology contributes to the questionability of results. Furthermore, evidence from interventional studies is very limited and the trials carried out report no beneficial results following alkali supplementation. Available literature suggests that poor acid load control may contribute to impaired insulin sensitivity and glucose homeostasis, but it is not sufficiently supportive to fully elucidate the issue and additional well-designed studies are clearly needed.

## 1. Introduction

Insulin resistance plays a pivotal role in the pathogenesis of multiple metabolic diseases, and it is considered the strongest predictor of diabetes development [1]. Vegetable-based diet consumption represents an effective strategy to control diabetes and insulinemia [2,3,4]. Conversely, the consumption of diets rich in protein and poor in vegetables may constitute a risk factor for the development of metabolic disturbances through the impairment of acid–base balance [5,6] and increasing attention has been focused on acid–base homeostasis in the aetiology of metabolic disorders including diabetes T2 [6]. Dietary intake can markedly influence the body’s acid–base balance [7,8]. Foods rich in animal-derived proteins increase acidic metabolic compounds, thereby disrupting metabolic homeostasis and increasing the risk of developing adverse conditions [5]. Accordingly, the health benefits gained though adherence to vegetables-based diets is due, at least in part, to the control of circulating metabolic acid levels [6].

Controlling extracellular pH within a specific range (7.35–7.45) [8] is critical for maintaining metabolic homeostasis. The net production of metabolic acids (acids different from CO_2_, which are excreted via renal system) represents the resultant of and all biochemical reactions occurring in the body and it is mostly constituted by the end-products of amino acids catabolism [6,8]. The totality of acid production is defined as net endogenous acid production (NEAP), which essentially reflects the net amount of acid excretion (NAE^§^ or RNAE). NEAP can be indirectly estimated using dietary data from protein intake and an index of base precursors from organic anions (e.g., potassium, which reflects vegetable consumption) [7] or other methods via potential renal acid load (PRAL)* calculation [9,10] (see Table 1 for details). The daily NAE in healthy adults ranges between 50 and 100 mEq/day (≈48 mEq/day) [6,11], and amino acid catabolism (sulphuric ammino-acids) is largely responsible for the acid load daily production and NAE increase [6,11]. Conversely, the catabolic pathways of certain plant-derived foods constituents such as citrate and malate tend to buffer free hydrogen ions (e.g., vegetables are characterized by low or negative values of PRAL) (Remer 2001), thereby, the regular consumption of fruit and vegetables and can help prevent the accumulation of circulating acidic compounds [8,12].

Accumulating evidence implicates the involvement of acid–base imbalance in the worsening of insulin’s glucose lowering effects [13,14,15]. In patients with end-stage kidney failure, both insulin sensitivity and glucose tolerance are impaired [16], whereas the correction of metabolic acidosis following bicarbonate treatment was shown to increase insulin sensitivity [17]. Experimentally-induced metabolic acidosis impairs glucose metabolism in humans by reducing cellular sensitivity to insulin [18]. Metabolic acidosis reduces the binding of insulin to its receptors in isolated adipocytes [19,20], alters the intracellular insulin signaling pathway [21], and increases production of the anti-insular hormone cortisol [22,23,24]. Elevated levels of serum lactate correlated with increased risk of diabetes T2 in the atherosclerosis risk in a community study [25], and insulin resistance and diabetes have been associated with lower urinary levels of citrate [26] and low urine pH. Collectively, these findings have led us to hypothesize that slight variation of pH caused by excessive dietary acid load impairs insulin sensitivity and glucose homeostasis.

In this respect, the present review is aimed to examine available information and relevant studies to elucidate a causal link and mechanism through which metabolic acid load affects insulin sensitivity and glucose metabolism. A thorough understanding of the issue will be clinically relevant to substantiate preventive dietary recommendations and ad hoc therapy development.

§ NAE (mEq/day) = [titratable acids* + NH_4_⁺] − [HCO_3_^−^] 24-h urine [7].

* The potential renal acid load (PRAL) accounts for the intestinal absorption rates of protein, potassium, calcium and magnesium and the dissociation of phosphate reflecting a greater “acid-forming potential” [8].

## 2. Metabolic Acidosis Disrupts Insulin Sensitivity

Although there is no conclusive evidence, several pathophysiological mechanisms exist to explain the association between metabolic acidosis and insulin resistance. Systemic infusion of lactate in rats was found to reduce insulin-stimulated glucose transport in muscle during a hyper-insulinemic euglycemic clamp [28]. Animal models of severe ketoacidosis exhibit insulin resistance [29,30] and adipocytes from these models were shown to express lower concentration of insulin receptor [29]. In diabetic rats, insulin sensitivity was shown to be directly proportional to pH. Ammonium chloride administration to normal rats or to mildly acidotic diabetic rats causes almost total loss of responsiveness to insulin probably due both to effects at the insulin receptor and direct effects on glycolysis [30]. Wittaker et al., demonstrated that reversing the acidemia of ketoacidotic rats via NaHCO_3_ infusion increases adipocyte insulin binding, whereas the infusion of ammonium chloride decreases medium pH and insulin binding to adipocytes due to a change in receptor concentration [20]. In cultivated myoblasts and adipocytes, mild metabolic acidosis diminishes the binding of insulin to its receptor and reduces the activation of intracellular insulin molecular pathway [20,21]. Moreover, acidosis reduces insulin-dependent inhibition of cellular proteolysis through downregulation of IRS and AKT phosphorylation [31]. Other physiological responses to metabolic acidosis, including increased cortisol levels, have been proposed as causative mechanisms for insulin resistance. Cortisol acts as anti-insular hormone, being capable of inhibiting insulin signaling in peripheral tissues, such as skeletal muscle and adipocytes [32,33]. Interestingly, receptors on visceral fat depots are more responsive to cortisol that subcutaneous, further highlighting a possible link between increased cortisol and glucose homeostasis impairment since visceral adipocytes dysfunction seems to be crucial for the development of insulin resistance [32,34,35]. Cortisol secretion is stimulated by low pH to increase the plasmatic clearance of excess hydrogen ions [36,37]. In this respect, Buehlmeier et al.—pooling results from randomized trials—observed an overall decrease of adrenal-glucocorticoids secretion following alkali supplementation [38], although the effects on cortisol levels was not specifically investigated. As cortisol acts as anti-insular hormone, the augmentation of its circulating levels as a result of mild-metabolic acidosis may account for worsening insulin sensitivity [39]. Of note, Esche et al. demonstrated that this correlation is also reproducible in healthy patients with higher acid load and blood pH in the nominal range [22]: screening 200 children in the Dortmund Nutritional and Anthropometric Longitudinally Designed (DONALD) the authors identified a positive correlation between acid load and cortisol concentration or its urinary markers. Furthermore, Maurer and colleagues reported a slight decrease in cortisol levels following supplementation with KHCO_3_ in healthy individuals [40]. Of note, cortisol levels and serum pH level both fell within the normal range prior to intervention. Some minor evidence substantiates the effect of acidosis on insulin-related mediators. For instance, low medium pH downregulates the expression of adiponectin [41], an insulin sensitizer, which modulates chronic inflammation and cell insulin signaling [42] and serves as predictor of insulin-resistance onset [43].

Leptin is a peptidic hormone involved in the regulation of glycaemic homeostasis being partly responsible for insulin peripheral effects [44,45]. Leptin was shown to reverse insulin resistance and diabetes via modulating adipocyte lipolysis/lipogenesis and hepatic gluconeogenesis probably via suppressing the hypothalamic-adrenal axis [45,46]. Leptin secretion was found to be downregulated in adipocytes exposed at low pH medium [47] and the administration of low doses (0.05–2 g/kg of body weight) of NaHCO_3_ in patients affected by renal-induced acidosis resulted in significant increase of serum leptin levels [48], results that lead to speculate that leptin deregulation, in the case of acidosis, can be implicated in the decrease of insulin sensitivity.

## 3. Muscle Metabolism and Lean Mass Are Influenced by Acid–Base Balance

Preservation of skeletal muscle mass and functionality is critical for glucose homeostasis [49,50] as it is the primary site of insulin-stimulated glucose uptake. Insulin sensitivity and β-cell functionality are dependent on muscle health, and a metabolically active muscular system has been shown to improve glycemia and insulin sensitivity in healthy and diabetic subjects [51]. Reduced muscle mass and strength is commonly associated with obesity and insulin resistance, in addition to other chronic metabolic perturbations, and abnormalities in insulin signal transduction have been associated with reduced activity of insulin-dependent glucose transport in skeletal muscles in diabetic patients [50]. To this end, muscle insulin-insensitivity accelerates muscle degradation by affecting insulin signaling [52], and end-stage kidney failure patients, suffering from mild metabolic acidosis, exhibit significant muscle loss compared with healthy subjects [16]. Conversely, alkali salt supplementation offsets the acidosis-induced muscle mass wasting [53]. Although the underlying mechanism is unclear, the finding that in cell models, proteasome activity increases upon pH lowering suggests augmented protein breakdown may occur [54,55,56]. Notably, the activation of ubiquitin-proteasome pathway is responsible for protein degradation in muscles of insulinopenic rats [57]. Protein and amino-acid degradation would provide energetic substrates and increase NH_3_ availability to promote hydrogen-ions disposal via kidneys [6]. In addition, the disruption of insulin-growth factor molecular signaling has been proposed as a possible mechanism for muscle loss as both insulin and insulin-like growth factors (IGFs) represent trophic mediators for muscle growth and maintenance [21,58,59]. IGFs exhibit molecular similarity to insulin and are capable of binding to the insulin receptor and its own receptor (IGFR) which share common signaling mechanisms [58]. Artificially-induced acidosis rapidly decreases IGF-1 levels in laboratory animals, and this effect is reversed following KHCO3 supplementation [60,61,62]. Moreover, metabolic acid load influences lean mass preservation. Using dual-energy X-ray absorptiometry (DEXA) measurements in adult women, Welch et al. reported a slight positive association between a more alkaline diet, lean mass, and muscle parameters [63]. A prospective cohort study conducted on approximately 3000 individuals found slower muscle mass wasting among persons over 65 with a lower NEAP (energy-adjusted estimated value) across four years [64]. Three interventional trials have investigated whether alkali supplementation may influence lean body mass status. A short-term supplementation with KHCO_3_ (60–120 mmol/day) for 18 days in 14 healthy postmenopausal women reduced urinary total nitrogen levels (−14.1 ± 12.3 g, *p* < 0.001) [65]. Similarly, two studies reported that KHCO_3_ was sufficient to reduce nitrogen excretion in middle-aged or older subjects [62,66] (see Table 2 for details). Since urinary nitrogen can reflect the rate of protein degradation in physiological conditions (assuming steady dietary protein intake, fecal nitrogen excretion negligible (<12%) and unaffected by alkali administration) [62], the authors suggest that supplementation may be effective for the preservation of muscle protein. Interestingly, in one of the investigations mentioned, supplementation with alkali salt was correlated with increased muscle performance (+70% of power measured with one rep at leg press) in women >50 year-old, whereas in men did not elicit any effect [66].

## 4. Association between Acid load and Glucose Homeostasis

Cross-sectional studies on large groups of individuals are likely to indicate a direct relationship between markers of acid load (or acid-imbalance) and insulin sensitivity or glycemic control. This is in accordance with the observation that consuming higher quantities of fruit and vegetables (low amount dietary acid load) reduces the risk of developing insulin resistance and diabetes [2,3]. Increased NAE, as well as other measures of mild metabolic acidosis (e.g., low serum bicarbonate, high anion gap, and low urinary pH [15,67,68]) have been associated with an increased risk of diabetes T2. In a large epidemiological investigation (NHANES), lower bicarbonate plasmatic levels and higher anion gap have been independently associated with decreased insulin-sensitivity in woman aged 30–55 [69]. Similarly, elevated plasma bicarbonate measured in 630 white, overweight women not suffering from metabolic conditions was associated with reduced risk of diabetes T2 (self-reported diagnosis) after adjusting for BMI, creatininemia, and history of hypertension [67]. Several incidence studies have been conducted on a large cohort of healthy patients to investigate the association between diabetes T2 or insulin resistance and markers of increased renal acid load. In the first study, a large sub-cohort of 66,450 female individuals from the E3N study (which has been followed-up for 14 years) was evaluated to estimate the incidence of diabetes T2. During follow-up, 1372 cases of diabetes T2 were validated though self-reported diagnosis (see Table 3). The result demonstrated that the highest quartile, according to acid load, was associated with a significant increase in diabetes T2 development compared to the lowest quartile (HR 1.56, 95% CI 1.29, 1.90) [15]. Of note, the association between NEAP and diabetes was found to be higher in normal weight women (BMI < 25 kg/m^2^) than in overweight/obese women. Kiefte-de Jong et al. [14] pooled results from three large epidemiological prospective studies conducted on healthy professionals (NHS, NHS2, and NPFS), and dietary data were obtained through FFQ in which both NEAP and PRAL were calculated. After adjusting for BMI, energy, and metabolic-variables, a positive correlation among individuals with higher acid load and incidence of diabetes was determined. Although these findings strongly suggest a positive correlation between acid load and diabetes T2 development, several investigations have reported conflicting results. In a cohort of individuals (27,809 men and 36,851women) aged 45–75, PRAL was found to be associated with the onset of diabetes T2 in young males (<50 year-old) but not females [70]. A further cross-sectional study by the same authors carried out on 1720 adult individuals (The Furukawa Nutrition and Health Study) substantiated the association between NEAP and both fasting insulin levels and HOMA-IR, but failed to demonstrate any correlation of acid load markers with HbA1c or fasting plasma glucose concentration [71]. Xu et al. [13] tracked 911 healthy men aged 70–72 for 18 years to assess the incidence of diabetes (115/911 cases) and its correlation with the dietary acid load. The study adopted glucose tolerance tests (GTT) and euglycemic–hyperinsulinemic clamp techniques to determine insulin sensitivity and β-cell functionality through IGI calculation

IGI = (insulin 30′ 2212 insulin 0′)/(glucose 30′ − glucose 0′).

Acid load was estimated by using both PRAL and NEAP based on a seven-day dietary diary. The study found no correlation between acid load and insulin resistance or β-cell dysfunction. However, the long period of follow up (which can bias the dietary data collected), the relative smallness of the sample size, and the specific age-group of participants enrolled (elderly) are significant confounding factors which can have affected the results achieved, accounting for the lack of consistency with other findings.

Bicarbonate supplementation was shown to be effective in offsetting acidosis in patients with end-stage kidney failure [17]. Evidence from interventional studies demonstrating an influence of supplementation on insulin or glucose homeostasis biomarkers is limited. Only two studies have been conducted to investigate whether lowering acid load via the administration of alkali could be beneficial for either insulin sensitivity or glucose metabolism. In a well-designed placebo-controlled study, Kozan et al. [72] estimated the effect of a pre-meal NaHCO_3_ bolus ingestion in 30 individuals with no reported metabolic disturbances in insulin sensitivity or plasma glucose. The authors found that bicarbonate ingestion offset the after-meal-pH lowering observed in the placebo group independent of glucose or insulin concentrations, thereby excluding any preventive effect on postprandial glycemia elicited by alkali supplementation [72]. Another placebo-controlled trial [73] was conducted on 153 middle-aged or older individuals not suffering from metabolic disturbances in which KHCO_3_ or NaHCO_3_ was administered for three months. No change in fasting glucose, serum insulin level, or HOMA-IR, was recorded in the bicarbonate-supplemented groups compared with controls (placebo/KCl supplemented).

## 5. Discussion

**Figure 1:**
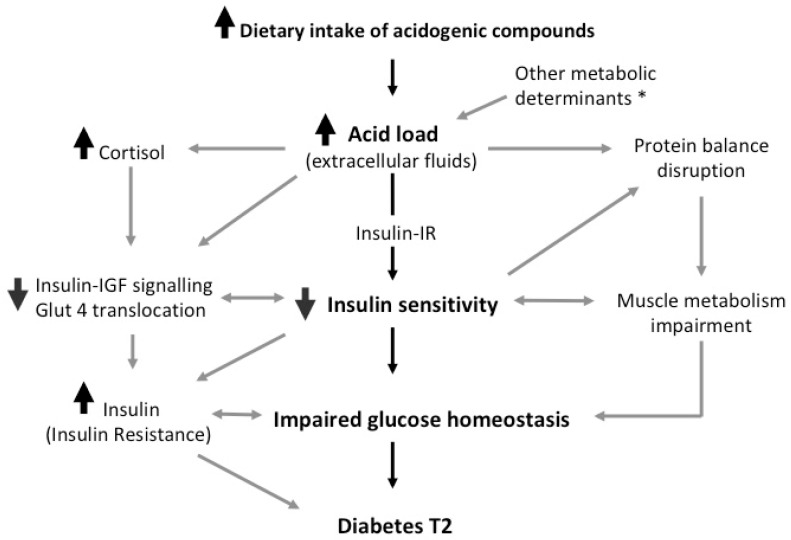
Synopsis of the possible underlying mechanism. Cortisol and other secondary mediators interfere with peripheral insulin activity via both impairing insulin receptor signal transduction and lowering the activation of intracellular insulin cascade [23,24]. The de-phosphorylation of the insulin receptor and key regulators, such as Akt [20,21], suppresses the translocation of GLUT4 in target tissues and downregulate the anabolic reactions propelled by insulin, including the inhibition of muscle protein breakdown [54,58,59]. This mechanism is likely to be independently promoted by pH imbalance secondary to acid load increase, and may account for the preservation of muscle mass when the acid load is low [63,64]. * Factors inducing the increase of acid load: catabolism end-products, metabolic conditions, kidney failure.

The data reviewed here strongly suggests an association between glucose metabolism and acid load biochemical markers. Findings which propose an active role of diet-induced acidosis in the regulation of insulin sensitivity are in accordance with experimental data showing that in models of metabolic acidosis, such as end-stage kidney failure, insulin sensitivity can be restored via alkali supplementation [17,53]. Dietary acid load may be modulated through specific dietary adjustments and the results discussed above are in line with the observation that plant-based diet consumption is an effective management for glucose-imbalance and metabolic disturbances [2,3]. In this sense, the health benefits exerted on diabetes and insulin-resistance achieved consuming vegetables-based diets can be also explained through the control of circulating metabolic acid levels [6]. As reported, a significant body of evidence supports the critical role of acidosis in the disruption of peripheral insulin activity through the interference with its receptor binding and intracellular insulin-signal transduction (see Figure 1).

On the other hand, the mechanisms driving the impairment of insulin function following augmented acid load outlined here are speculative and the findings reviewed here prohibit us from discerning the causes of metabolic acid load. Several other underlying metabolic conditions independent of diet composition can influence acid load, and other biochemical mechanisms regulating pH may underlie the consumption of specific diets. In this respect, Mandel et al. [67] found that adjusting primary results according to animal-derived protein and potassium intake resulted in no attenuation in the association between plasma bicarbonate and diabetes, suggesting that these underlying mechanisms may be independent of dietary acid load. Furthermore, available evidence does not allow for inference on the relationship cause and effect: metabolic disturbances such as diabetes itself can affect pH status and the production of acid species [74,75] by affecting metabolic reactions and kidney functionality [76,77].

Several other limitations affecting the studies reviewed can be highlighted, which can also help the interpretation of results as well as lay the basis for further investigations.

### 5.1. Parameters Estimation

The degree of insulin sensitivity is often quantified through HOMA-IR determination [78], which is dependent on the reliability of primary data. In addition, HOMA-IR constitutes an indirect estimation methodology which needs to be carefully interpreted in subjects with low BMI, low β-cell function, and high fasting glucose levels or older individuals with impaired glucose tolerance [79,80].

Calculated NEAP is an indirect measure adopted across the studies reviewed her, although the standard method to quantify acid load is NAE as elsewhere reported [27]. Adopting NEAP (or PRAL) as an indirect estimation of acid load is justified since validated measures were shown to reflect urinary pH in the correlation with diabetes T2 [81]. NEAP calculation is based on the assumption of a theoretical metabolic steady state, which does not apply to conventional nutritional studies nor to the investigations discussed here [27]. Although the estimation of PRAL and NEAP from dietary recalls are considered valid reflections of real renal net acid excretion, some limitations can emerge when underreporting bias occurs, such as in obese patients [82]. In the E3N-EPIC study [15], the correlation with acid load and diabetes was found to be weaker in obese and overweight subject, supporting this instance. Moreover, Frassetto et al. noticed that indirect estimation formulas can underestimate NAE in diabetes T2 affected subjects undergoing dietetic regimens with different degrees of dietary acid load [83]. Additional endocrine and metabolic determinants may interfere with endogenous acid generation and buffering [84]. For example, hormone-replacement therapy was shown to influence acid–base balance [85], suggesting that sexual hormones can be modulatory on acid load. Differences in results, however, may also occur because PRAL and NEAP estimation from dietary content have been validated against acid load measured in 24-h urine samples only in western countries [7,8].

Since metabolic acid disposal is regulated by kidneys, renal clearance impacts acid–base balance [76,77], and kidney disturbances can cause significant increases in NEAP values [27]. Hence, the relationship between dietary acid load and diabetes T2 can be more pronounced in participants with impaired kidney function resulting from altered hemodynamic adaptation to high acid load [86]. In the case of altered renal clearance, NEAP may impact results by underestimating the actual value of plasmatic acid load. In addition, surrogate insulin sensitivity indexes, such as the HOMA-IR, can be influenced by renal retention of these metabolites in the setting of kidney failure [77]. Of note, diabetes itself can modify kidney hemodynamic and electrolyte imbalance [74], and subclinical or undiagnosed conditions may be present before diagnosis, especially in long-term studies. By contrast, NEAP assessment through NAE measurement in diabetic or pre-diabetic patients may be misleading since diabetes influences urinary pH and overly acidic urine in patients with diabetes T2 was shown to persist even after controlling for dietary factors [87].

### 5.2. Nutritional and Metabolic Confounders

Focus on single determinants can be challenging due to extraneous factors including the presence of nutritional and metabolic confounders and lifestyle habits such that data can be misleading, especially in the case of large epidemiological studies. For instance, the presence of diabetes (pre-diabetes or undiagnosed diabetes) constitutes a factor promoting the increase of acid load as noted above. Population groups at risk for metabolic disturbances are often homogeneous in the context of certain variables and exhibit intrinsic confounders: individuals positive for diabetes are likely to have higher prevalence of metabolic syndrome, poor exercise habits, lower daily fiber intake, and higher intake of trans fats and high-glycemic index foods [67].

BMI and overall energy intake can act as confounders by influencing insulin sensitivity and glucose homeostasis. In addition, differential adipose tissue deposition (visceral or abdominal) can be pivotal to sustain the development of such metabolic disturbances [35]. For instance, two studies reported that the association between markers of high acid load and insulin resistance was more pronounced in participants with higher BMI [69,71]. Apart from this, a number of macro and micro-nutrients such as protein or specific types of lipids can independently modulate insulin sensitivity [88]. Baudrand et al. found that high sodium diets are associated with increased cortisol secretion and insulin resistance [89]. Animal-derived foods increase circulating micro-elements, including salt and iron, which have been suggested to impair metabolic homeostasis [6,11,90,91]. Although non-conclusive, some evidence has reported that either circulating iron or markers of iron stores can inversely correlate with insulin sensitivity and glycemic control [92,93,94]. Importantly, ketogenic diets and high-fat diets demonstrated to be effective for glycemic control in diabetic subjects [95,96] clearly affect acid–base balance, suggesting that the detrimental effect promoted by the excessive concentration of acid compounds may be less significant.

One of the major concerns of vegetable-based diets is the role of dietary fiber. As noted above, vegetable-based diets are low acid producing diets, and high fiber consumption constitutes a dietetic strategy to control body weight and help prevent the onset of diabetes and insulin resistance [2,3,4,5,97]. Fiber intake stimulates satiety and it is correlated with decreased post-prandial insulin demand [98] by modulating glucose absorption and regulating gastrointestinal hormone-like peptides secretion [99,100] such as glucagon-like peptide 1 [101]. Hence, epidemiological studies reporting amelioration of insulin sensitivity following vegetable-based diet consumption should be interpreted in the context of effects elicited by fiber on energetic metabolism and glucose homeostasis.

### 5.3. Investigation Methodology

Several of the cohort studies discussed above [14,15,67,69] report data from investigations in which groups of individuals were homogeneous with regard to gender and profession. The inclusion of homogeneous individuals in terms of age, sex, ethnicity, and geographical distribution may generate poor NEAP variability. Factors such as age and gender are likely to influence results, and the homogeneity of the sample population may limit the generalizability of results. For instance, in the prospective cohort study of Akter et al., the authors noticed that the correlation of acid load with diabetes was different for men and women (with no correlation found in women) and, after age stratification, the resulting correlation was only apparent in younger individuals [70]. The study of Xu et al., testing only older men (aged 70–71) [13], showed that higher NEAP corresponded to increased insulin sensitivity. An inverse relation occurring in the elderlies was also reported by Akter et al. [70]. Of note, the lack of association observed among older individuals may be affected by the presence of background risk factors for diabetes—such as hyperlipidemia, high blood pressure, and altered glucose tolerance [102]—which can bias results. In addition, the adherence to healthy diets is plausibly linked to healthier lifestyle-related habits and factors—such as obesity, smoking, physical activity, and education exposure—which influence insulin sensitivity and diabetes incidence as demonstrated elsewhere [103]. In this respect, it is notable that some of the most relevant prospective studies used as database (NAHNES, NHS, HPFS, E3N) have been carried out on white individuals with medium-high education status (E3N enrolled school teachers) and/or health-knowledge (health professionals), which could compromise the strength of the evidence. In addition, self-reported diagnosis of diabetes [15,71] may underestimate its actual incidence in the study population [104]. Moreover, since individuals are typically followed-up with for several years to test diabetes incidence, it is likely to presume that their dietary habits (or lifestyle habits) underwent modifications over time. Minor limitations such as the different methodologies for NEAP across studies [7,9,10] and the difficulty in discerning between diabetes T1 and T2 when cohort studies data are retrospectively analyzed can be also highlighted.

Finally, there is a significant shortfall of interventional studies on this topic as to substantiate observational evidence. The only two available studies have recorded no significant metabolic effect following supplementation. Of note, the only reported study on prolonged supplementation [73] was not specifically designed to assess glucose metabolism variables and no direct measurement of NAE was assessed. Importantly, future studies should screen participants based on dietary habits in order to minimize possible dietary confounders and correctly randomize subjects for treatment.

## 6. Conclusions

The control of metabolic acid load appears to be relevant for global metabolic health and may be one of the variables promoting insulin sensitivity and glucose homeostasis impairment in the case of sub-optimal nutritional habits. Furthermore, it could be promising in the light of potential therapy based on alkaline supplementation to enhance glycemic control in diabetic or insulin-resistant patients.

Results from epidemiological studies are suggestive but not conclusive due to multiple experimental confounds. The shortfall of interventional studies and the poor knowledge of the underlying mechanism contribute to weakening existing evidence. Taken together, the limitations discussed above underscore the extreme difficulty of achieving clear data. At present, all results suggest that the poor control of dietary acid load can be an additive mechanism underlying glucose metabolism impairment. Therefore, it might be speculated that the beneficial effects gained from vegetable-based diets consumption are partly due to the restraint of dietary acid load.

Future investigations are needed to support existing evidence as well as to better elucidate the issue. Experimental analysis should be specifically designed to rule out any potential confounding factors as well as minimize the impact of all the extra variables which negatively affect current studies.

## Figures and Tables

**Table 1:** Indirect estimation of NEAP, according to available equations [27]

Author	Equation
Frassetto et al.	NEAP (mEq/day) = [0.91 × protein (g/day) − 0.57 × potassium (mEq/day)] + 21 NEAP (mEq/day) = [54.5 × protein (g/day)/potassium (mEq/day)] − 10.2
Remer et al.	NEAP_est_ (mEq/day) = PRAL * + OA_est_
Sebastian et al.	NEAP_est_ (mEq/day) = PRAL + OA_est_ §

* PRAL (mEq/day) = 0.49 × protein (g/day) + 0.037 × phosphorus (mg/day) − 0.021 × potassium (mg/day) − 0.026 × magnesium (mg/day) − 0.013 × calcium (mg/day) [27]. OA_est_ (mEq/day) = body surface area × 41/1.73 [27]. § OA_est_ (mEq/day) = 32.9 + 0.15 × [sodium (mg/day) + potassium (mg/day) + calcium (mg/day) + magnesium (mg/day) − chloride (mg/day) – phosphorus (mg/day)].

**Table 2:** Human studies showing the impact of metabolic acid load status on muscle mass.

Author	Subjects ^1^	Age (year)	Study Type	Variables Measured	Results	Duration/Design
Welch et al., 2013 [63]	2689 women	18–79	Cross-sectional	Fat mass Fat-free mass PRAL	Lower quartile of PRAL correlates with a less preserved fat-free mass	-
Chan, 2015 [64]	3122 men and women	>65	Cohort Prospective	Axial muscle mass Energy-adjusted NEAP	Participants in the highest quartile of energy-adjusted estimated NEAP lost significantly more muscle mass than those in the lowest	4 years
Frassetto et al., 1997 [65]	14 postmenopausal women	51–77	Intervention clinical trial	NAE Nitrogen excretion	Alkali supplementation reduced NAE and nitrogen excretion	18 days 60–120 mmol/day of KHCO_3_
Ceglia et al., 2009 [62]	19 men and women	54–82	Double-blind, randomized, placebo-controlled study	IGF-I Urinary nitrogen Urinary calcium	KHCO_3_ reduced the rise in urinary nitrogen excretion that accompanied an increase in protein intake	90 mmol/day of KHCO_3_ 41 days ^2^
Dawson-Hughes, 2010 [66]	71 men 91 women	>50	Double-blind, placebo-controlled trial	NAE Nitrogen excretion Muscle power Training endurance	KHCO_3_ reduced NAE and nitrogen excretion. In women, bicarbonate increased double leg press power at 70% one repetition maximum by 13%	67.5 mmol/day of KHCO_3_ for 3 months

^1^ All studies are performed on healthy subjects with no metabolic conditions. ^2^ KHCO_3_ or placebo with a 16-day phase-in and two successive 10-day diets at low (0.5 g/kg) or high (1.5 g/kg) protein in randomly assigned with a five-day washout period between diets.

**Table 3:** Synopsis of the studies investigating the correlation of markers of acid load with insulin resistance and diabetes *.

Author	Subjects ^1^	Age (year)	Study Type	Variables Measured	Results	Duration/Design
Farwell et al., 2008 [69]	1496 women	>12	Cross-sectional	HCO_3_^−^ Insulin resistance via both HOMA-IR and MFFM	Lower anion gap and bicarbonates correlate with increased insulin resistance	-
Mandel et al., 2012 [67]	630 (and 730 controls) (nurses)	30–55	Prospective nested case-control	HCO_3_^−^ Self-Reported T2D diagnosis	Lower bicarbonates correlate with increased diabetes T2 incidence	10 years
Fagherazzi et al., 2014 [15]	66, 485 women (teachers)	mean 53	Cohort retrospective	PRAL NEAP Self-reported T2D ^2^	Highest PRAL-NEAP quartile shows higher incidence of diabetes T2 compared to lowest	14 years
Kiefte-de Jong et al., 2016 [14]	67,433 women ^3^ 84,310 women 35,743 men	30–55 25–42 40–75	Cohort retrospective	PRAL NEAP A:P ratio ^4^ T2D	Highest PRAL-NEAP and A:P quartile shows higher incidence of diabetes T2 compared to lowest	24 years
Akter, et al., 2016 [71]	1536 men 169 women (manifacture workers)	19–69	Cross-sectional	PRAL, NEAP HOMA-IR HOMA-β HbA1c Fasting glucose	PRAL and NEAP associated with HOMA-IR ^5^ NEAP positively associates with HOMA-β No association with fasting glucose and HbA1c	-
Akter, et al., 2016 [70]	27,809 men 36,851 women	45–75	Cohort retrospective	PRAL, NEAP Self reported T2D diagnosis	Only PRAL associates with T2D incidence in men < 50 year-old	10 years
Xu et al., 2014 [13]	911 men	70–71	Cohort Prospective	PRAL, NEAP Insulin resistance T2D ^6^	No association of PRAL-NEAP with insulin sensitivity, β-cell function or diabetes incidence	18 years
Kozan et al., 2017 [72]	20 men 10 women	24–44	Placebo-controlled, crossover trial	C-peptide Insulin Fasting glucose Glucose (0–180′) GLP-1	No effect of NaHCO_3_ on postprandial insulin, plasma glucose, C-peptide and GLP-1 compared to placebo	0-180 min - placebo - NaHCO_3_ (1680 mg)
Harris et al., 2010 [73]	153 men and women ^6^	>50 mean 64	Randomized, placebo-controlled trial	HOMA-IR Insulin Fasting glucose	No effect of either NaHCO_3_ or KHCO_3_ on insulin, plasma glucose and HOMA-IR compared to placebo	84 days - placebo or 67.5 mmol/day of - KCl - NaHCO_3_ - KHCO_3_

* Plasma bicarbonate was included as a marker of metabolic acidosis; ^1^ Healthy subjects in all studies reported, with no metabolic conditions at baseline; ^2^ Participants were also considered diabetic if reporting elevated glucose concentration (fasting glucose ≥ 7.0 mmol/L or random glucose ≥ 11.1 mmol/L), treatment with diabetes drugs, and/or fasting glucose or HbA1c ≥ 7%. (53.0 mmol/moL); ^3^ Participants were all health professionals; ^4^ animal protein-to-potassium ratio; ^5^ In the stratified analyses, positive associations were confined to subjects with lower BMIs (<23 kg/m^2^) (P 0.03 and 0.01 for PRAL Pand NEAP, respectively); ^6^ Euglycemic–hyperinsulinemic clamp technique and the GTT to determine insulin sensitivity and β-cells function (through the calculation of IGI). Diabetes incidence was defined using fasting concentration of glucose (fasting plasma glucose ≥ 7.0 mmol/L) or the use of glucose-lowering medication; ^7^ All menopausal women.
